# Onchocerciasis transmission in Ghana: the human blood index of sibling species of the *Simulium damnosum* complex

**DOI:** 10.1186/s13071-016-1703-2

**Published:** 2016-08-05

**Authors:** Poppy H. L. Lamberton, Robert A. Cheke, Martin Walker, Peter Winskill, J. Lee Crainey, Daniel A. Boakye, Mike Y. Osei-Atweneboana, Iñaki Tirados, Michael D. Wilson, Anthony Tetteh-Kumah, Sampson Otoo, Rory J. Post, María-Gloria Basañez

**Affiliations:** 1 London Centre for Neglected Tropical Disease Research, Department of Infectious Disease Epidemiology, School of Public Health, Faculty of Medicine (St Mary’s campus), Imperial College London, Norfolk Place, London, W2 1PG UK; 2 Natural Resources Institute, University of Greenwich at Medway, Central Avenue, Chatham Maritime, Chatham, Kent ME4 4 TB UK; 3 MRC Centre for Outbreak Analysis and Modelling. Department of Infectious Disease Epidemiology, School of Public Health, Faculty of Medicine (St Mary’s campus), Imperial College London, Norfolk Place, London, W2 1PG UK; 4 Instituto Leônidas e Maria Deane, Fundação Oswaldo Cruz, Rua Terezina 476, Adrianopolis, AM 69057-070 Manaus, Brazil; 5 Noguchi Memorial Institute for Medical Research, University of Ghana, Legon, PO Box LG581, Accra, Ghana; 6 Department of Environmental Biology and Health, Water Research Institute, Council for Scientific and Industrial Research, PO Box M32, Accra, Ghana; 7 Department of Vector Biology, Liverpool School of Tropical Medicine, Pembroke Place, Liverpool, L3 5QA UK; 8 Ghana Health Service, P.O. Box MB-190, Accra, Ghana; 9 School of Natural Sciences and Psychology, Liverpool John Moores University, Byrom Street, Liverpool, L3 3AH UK; 10 Present address: Institute of Biodiversity, Animal Health and Comparative Medicine; Wellcome Trust Centre for Molecular Parasitology, University of Glasgow, Glasgow, G12 8QQ UK

**Keywords:** Human blood index, Host choice, *Simulium damnosum* (*sensu lato*), Host-seeking vectors, Ovipositing vectors, *Onchocerca volvulus*, Vector abundance

## Abstract

**Background:**

Vector-biting behaviour is important for vector-borne disease (VBD) epidemiology. The proportion of blood meals taken on humans (the human blood index, HBI), is a component of the biting rate per vector on humans in VBD transmission models. Humans are the definitive host of *Onchocerca volvulus*, but the simuliid vectors feed on a range of animals and HBI is a key indicator of the potential for human onchocerciasis transmission. Ghana has a diversity of *Simulium damnosum* complex members, which are likely to vary in their HBIs, an important consideration for parameterization of onchocerciasis control and elimination models.

**Methods:**

Host-seeking and ovipositing *S. damnosum* (*sensu lato*) (*s.l.*) were collected from seven villages in four Ghanaian regions. Taxa were morphologically and molecularly identified. Blood meals from individually stored blackfly abdomens were used for DNA profiling, to identify previous host choice. Household, domestic animal, wild mammal and bird surveys were performed to estimate the density and diversity of potential blood hosts of blackflies.

**Results:**

A total of 11,107 abdomens of simuliid females (which would have obtained blood meal(s) previously) were tested, with blood meals successfully amplified in 3,772 (34 %). A single-host species was identified in 2,857 (75.7 %) of the blood meals, of which 2,162 (75.7 %) were human. *Simulium soubrense* Beffa form, *S. squamosum* C and *S. sanctipauli* Pra form were the most anthropophagic (HBI = 0.92, 0.86 and 0.70, respectively); *S. squamosum* E, *S. yahense* and *S. damnosum* (*sensu stricto*) (*s.s.*)*/S. sirbanum* were the most zoophagic (HBI = 0.44, 0.53 and 0.63, respectively). The degree of anthropophagy decreased (but not statistically significantly) with increasing ratio of non-human/human blood hosts. Vector to human ratios ranged from 139 to 1,198 blackflies/person.

**Conclusions:**

DNA profiling can successfully identify blood meals from host-seeking and ovipositing blackflies. Host choice varies according to sibling species, season and capture site/method. There was no evidence that HBI is vector and/or host density dependent. Transmission breakpoints will vary among locations due to differing cytospecies compositions and vector abundances.

**Electronic supplementary material:**

The online version of this article (doi:10.1186/s13071-016-1703-2) contains supplementary material, which is available to authorized users.

## Background

Vector biting behaviour is crucially important in the epidemiology and transmission dynamics of vector-borne diseases (VBDs). Understanding vector blood-feeding patterns can assist in comprehending the effectiveness and suitability of different vector control strategies [1] and improve the accuracy of transmission dynamics models [2]. For example, in areas where malaria transmission is mediated predominately by the inherently anthropophagic vector *Anopheles gambiae* (*sensu stricto*) (*s.s*.), classical zooprophylaxis (the use of non-human blood hosts to divert vector biting) [3] is unlikely to have a significant impact on vectorial capacity (the potential for infection transmission). In another example, because *A. arabiensis* is exophagic and has post-prandial exophilic tendencies, treatments of cattle with insecticides may be an effective control option [1].

The proportion of blood meals taken on humans, also known as the human blood index (HBI) [4], is a component of the biting rate per vector on humans, which is multiplied by the vector to human host ratio to provide the contact rate from vectors to humans in transmission models of VBDs. The vector to human host ratio (the size of the vector population divided by the size of the human host population, i.e. the number of vectors per person under an assumption of homogenous biting) is rarely measured separately. However, if human population densities and HBI values were known, it would be possible, at least in principle, to estimate total vector population abundance [2]. The HBI and the (parous) vector to human host ratio have been used as separate variables in a modelling investigation of the impact of climate change on populations of simuliid vectors of human onchocerciasis [5], but a greater understanding of how they vary among vector species is a pressing need.

The London Declaration on Neglected Tropical Diseases (NTDs) [6] and the World Health Organization’s (WHO) NTDs Road Map [7] have set goals for the elimination of human onchocerciasis in selected African countries by 2020. Precisely where and in what time horizon this goal can be achieved with current ivermectin distribution strategies (once or twice per year) depends on, among other things, detailed understanding of transmission across a range of ecological and epidemiological settings. An important determinant of the feasibility of reaching elimination within reasonable timelines, as set out by the WHO NTDs Road Map [7], is the level of onchocerciasis endemicity, determined by the prevalence and community load of *Onchocerca volvulus* microfilariae [8]. In order to set starting values for these parasitological indicators when running model simulations, the annual biting rate (ABR) of blackfly vectors becomes an important input variable. In turn, this hypothetical ABR depends on assumptions made regarding the HBI. Stolk et al. [8] fixed the HBI of the savannah members of the *Simulium damnosum* (*sensu lato*) (*s.l.*) complex at 0.96 in their comparison of the ONCHOSIM and EPIONCHO transmission models in order to better align input ABR values between the two models. A high value of the HBI translates into a more difficult to achieve transmission breakpoint in the deterministic framework of EPIONCHO [8–10]. However, this assumption needs to be examined to improve current parameterizations of such models [8, 10]. In particular, it is important to understand how the relative mix and vectorial capacity of blackfly vector species vary spatially (among transmission foci) and temporally (seasonally) as well as with host abundance [11]. Such understanding will also help to identify (i) the best timing of ivermectin treatment [12], (ii) when and where mass drug administration (MDA) of ivermectin could be halted without an unacceptably high risk of infection recrudescence, (iii) foci that could benefit from addition of antivectorial interventions, and (iv) vector trapping techniques to control vector populations and/or to monitor current transmission levels [13, 14].

The *Simulium damnosum* complex has approximately 60 sibling species and cytoforms [15, 16], which differ in their geographical distribution, ecological features, degree of anthropophily (attraction to humans) and anthropophagy (propensity to feed on humans), and vector competence and vectorial capacity for *O. volvulus* [10, 13, 15, 17, 18]. Understanding variations in HBI is important in human onchocerciasis, because although the definitive hosts of *O. volvulus* are humans (i.e. the infection is not a zoonosis), the simuliid vectors are known to feed on a range of blood hosts including cattle, turkeys and chickens [19–22] (and see Table 1 of [23] for a full list, which includes camels, elephants and giraffes). The argument is that parasites can be removed from the human transmission cycle by persistent biting of blackflies on non-human animals. In Cameroon, cattle herds have acted to divert *S. damnosum* (*sensu stricto*) (*s.s.*) blackflies from human to cattle blood meals [24], acting as a zooprophylactic effect in addition to annual MDA with ivermectin. Blackflies’ relative attraction to cattle is of even greater interest due to potential immunological interactions between *O. volvulus* and its closest phylogenetic relative, the cattle parasite *O. ochengi*. Humans bitten by blackflies carrying infective larvae of *O. ochengi* may exhibit a protective immunological response due to cross-reactivity between *O. ochengi* and *O. volvulus* [25].

**Table 1:** Survey of human and domestic animal population sizes and wild bird diversity in study villages, Ghana

Region	Village	No. of people (*H*)			No. of domestic animals											No. of species of wild birds
		Children (0–18 years)	Adults (≥18 years)	Total	Chickens	Ducks	Cattle	Sheep	Goats	Cats	Dogs	Pigs	Total birds	Total mammals (*M*)	*M*/*H*	
Brong-Ahafo	Asubende	46	62	108	321	23	75	22	52	13	3	0	344	165	1.53	33
	Agborlekame	nd	nd	nd	nd	nd	nd	nd	nd	nd	nd	nd	nd	nd	na	41
Volta	Asukawkaw Ferry	2,342	3,117	5,459	5,387	593	793	1,640	1,610	962	85	617	5,980	5,707	1.05	35
	Dodi Papase	2,178	3,076	5,254	5,234	653	0	1,071	2,285	275	204	0	5,887	3,835	0.73	50
	Pillar 83	76	112	188	358	3	0	59	68	13	22	0	361	162	0.86	61
Western	Bosomase	90	98	188	457	2	0	24	81	14	20	0	459	139	0.74	31
Ashanti	Gyankobaa	132	142	274	294	0	0	32	115	8	40	0	294	195	0.71	55

*Abbreviations:* *H* human host density, *M* non-human (domestic) mammal density, *M/H* non-human mammal to human host ratio, *nd* not determined, *na* not applicable

However, challenges exist when attempting to obtain unbiased HBI estimates, such as difficulties in interpreting results from host-dependent [22] and host-independent samples; difficulties in obtaining post-engorgement resting blackflies [21, 26], low specificity of Enzyme-linked immunosorbent assay (ELISA) techniques; limitations (to the specific host species tested) of anti-sera and ELISA studies [27, 28], and low blood meal DNA concentrations [29] in host-seeking and ovipositing flies.

In this study we focus on Ghana, firstly because this country has a diversity of *S. damnosum* complex members [30], which differ in their biting, parity and infection rates according to species, spatial distribution and temporal dynamics [13, 18], and therefore we expect the HBI also to vary across these dimensions; secondly, because we have documented persistent onchocerciasis transmission despite previous history of vector control and current ivermectin treatment [18] (and F. B. D. Veriegh, personal communication), and thirdly, because the adoption of a twice-yearly frequency of ivermectin treatment may not assuage concerns of sub-optimal responses to ivermectin [31]. It is, therefore, imperative to refine structural and parametric assumptions of onchocerciasis transmission models with which to assess the feasibility of onchocerciasis elimination by 2020 [8, 32]. The objectives of this study were to: (i) establish the range of blood hosts on which sibling species of the *S. damnosum* complex feed; (ii) understand how HBI varies among sibling species, locality, season and trapping method; (iii) report on the suitability of host-dependent and host-independent catching techniques for HBI determination; (iv) explore the suitability of molecular methods for blood meal origin determination (given the difficulty in obtaining freshly engorged, resting blackflies); (v) estimate the vector population size and the number of vectors per human using field-derived biting rates, recorded host population sizes and molecularly determined HBI; (vi) test the hypothesis that the HBI may be vector and/or host density dependent [2, 11], and (vii) provide data of epidemiological importance for improved parameterization of onchocerciasis transmission models.

## Methods

### Ethical clearance

Ethical clearance was obtained from the Imperial College Research Ethics Committee (ICREC_9_1_7) and the Institutional Review Board of the Noguchi Memorial Institute for Medical Research, University of Ghana (IRB:0001276, 006/08-09). No tissue samples were taken from human subjects; however, local villagers and elders assisted with blackfly collections. Signed informed consent was obtained from all individuals involved after detailed explanations about the study in their local languages. Participating individuals were not at an increased risk of exposure, nor were human samples obtained for diagnosis, therefore no treatments were offered. However, all participants were receiving ivermectin as part of the national programme following appropriate (annual or biannual) schedules according to the Ghana Health Service strategy [33].

### Study area

**Fig. 1:**
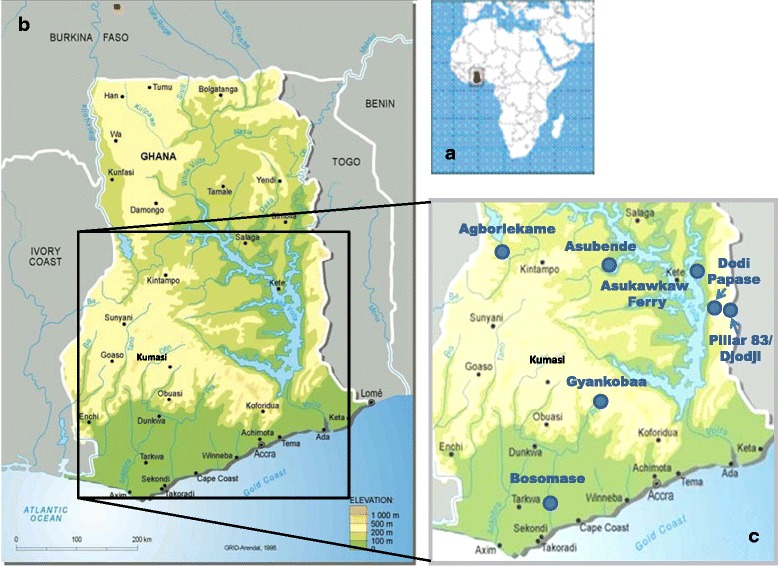
Location of the seven study sites in Ghana. **a** Map of Africa showing the location of Ghana. **b** Map of Ghana showing the general location of the study area in the bottom half of Ghana. **c** Location of the study villages: Asubende, Agborlekame, Asukawkaw Ferry, Dodi Papase; Djodji/Pillar 83; Bosomase and Gyankobaa

Site selection, geography and key simuliid species have been described in detail elsewhere [13, 18, 30]. Briefly, blackfly collection was conducted in seven villages within four regions of Ghana: Asubende (08°01'01.4"N, 00°58'53.8"W) and Agborlekame (08°14'04.0"N, 2°12'23.2"W) in the Brong-Ahafo Region; Asukawkaw Ferry (07°40'55.9"N, 00°22'16.0"E), Dodi Papase (07°43'22.5"N, 00°30'38.3"E) and Pillar 83/Djodji (07°42'20.3"N, 00°35'21.5"E) in the Volta Region (Pillar 83 is the village on the Ghanaian side of the river Wawa, which forms the border and is known as the Gban-Houa in Togo, opposite the former Onchocerciasis Control Programme in West Africa (OCP) catching site of Djodji in Togo); Bosomase (05°10'44.7"N, 01°36'23.1"W) in the Western Region, and Gyankobaa (06°20'12.4"N, 01°16'11.3"W) in the Ashanti Region (Fig. 1). A pilot study was conducted at Bosomase in January–February 2006 to assess the efficacy of Bellec traps (see below) as a fly collection method, and to test the performance of DNA amplification methods for the determination of blackfly species, infection status and blood meal origin. The main sample collection took place during one wet season, 23^rd^ July–5^th^ September 2009, and two dry seasons, 14^th^ February–28^th^ March 2010 and 30^th^ January–5^th^ March 2011. Villages were visited and samples were collected for up to five consecutive days per site per trip. Not all sites were successfully sampled during each period due to weather conditions and variability in blackfly population abundance. These villages varied in vector species composition and human population size.

### Household surveys

People and their domestic animals were surveyed in the study villages once at each sampling site after having the study explained and permission granted. The number of children (those aged < 18 years), adults (≥ 18 years), their cattle and other domestic animals such as chickens, ducks, sheep, goats, cats, dogs, pigs, and any other animals were recorded. All houses were surveyed at the villages of Asubende, Pillar 83, Bosomase and Gyankobaa. In Asukawkaw Ferry and Dodi Papase, information on the total number of houses in the whole town was gathered, and a stratified subsample was obtained of approximately every third house visited. Agborlekame was not surveyed, due to limited fly collection success and time constraints.

### Wild bird and mammal surveys

At each location a walking route of approximately 1 km was followed at least twice per sampling period, once in the early morning and once at dusk. Any tracks, sightings or hearings of wild animals were recorded. A consistent transect was difficult to achieve as all paths were constantly in use by local villagers with potential animal tracks often covered over.

### Blackfly sample collection

**Fig. 2:**
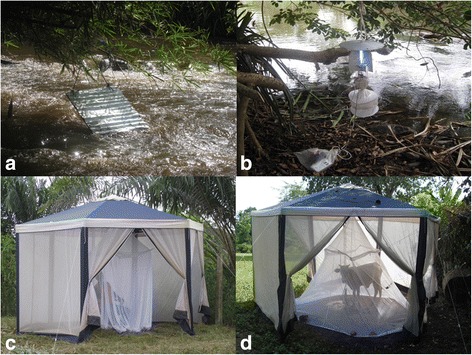
Methods used to obtain host-independent (**a**, **b**) and host-dependent (**c**, **d**) adult female blackfly samples. **a** Bellec (sticky) trap situated above rapids. **b** Monks Wood (light) trap placed near presumed breeding sites. **c** Human-baited tent. **d** Cow-baited tent. **a** and **b** illustrate traps to collect ovipositing flies; **c** and **d** depict methods to obtain host-seeking flies

Blackfly larvae and pupae, and adult host-seeking (host-dependent) and adult ovipositing (host-independent) blackfly collection methods for this study have been described in detail elsewhere [13, 18, 30]. Briefly, larvae and pupae, used to aid species identifications at a village level, were collected and stored in Carnoy’s solution, with larvae used to make and identify chromosomal preparations [30]. Host-seeking blackflies were caught from 7 am to 6 pm every collection day using a) human-baited tents (where the humans were protected from bites by a mosquito net within a larger catching tent), b) cattle-baited tents (with the cow protected from biting within a mosquito net inside a tent), and c) OCP standard vector-collector methods, the latter with an individual sitting with the lower half of their legs exposed collecting all blackflies which landed on them. Ovipositing blackflies were collected using a) sticky Bellec traps and b) UV Monks Wood light traps (Fig. 2).

### Blackfly species identification

Morphological and molecular fly identifications and dissections of host-seeking and ovipositing blackflies have been described previously [13, 18] and were performed on nearly all collected flies. The colour of the fore-coxae used by some authors [34, 35] to separate *S. damnosum* (*s.s*.) from *S. sirbanum* is unreliable since many individuals of both species with either dark or pale fore-coxae have been noted, especially in the eastern parts of the former OCP, and therefore these two species were not split by definitive identification and are referred to as *S. damnosum* (*s.s*.)/*S. sirbanum*. The abdomens from all collected flies were individually stored in absolute ethanol and used for the molecular identification of vector species, *Onchocerca* spp. larvae and host blood meal.

### Molecular blood meal analysis

Abdomens were individually removed from ethanol and rinsed in sterile water. They were then placed in individual 1.5 ml Eppendorf tubes in Qiagen lysis buffer and proteinase k, and macerated by hand using a sterilised plastic pestle. Samples were then incubated overnight at 56 °C. DNA was extracted using the Qiagen DNeasy® Blood and Tissue kit as per protocol and frozen at -20 °C. Frozen samples were then shipped to Eurofins (Germany) (https://www.eurofinsgenomics.eu/) for blood meal profiling [36].

### Statistical analyses

Statistical analyses were performed using R [37]. The probability of detecting a blood meal, referred to as the amplification success rate, was calculated as the proportion of flies from which (non-*Simulium*) DNA was successfully extracted. The probability of detecting a human blood meal (i.e. the HBI) was calculated as the number of flies with human blood meals as a proportion of the number of flies with any blood meal detected. For descriptive, univariate purposes, these were calculated from flies in which only one blood meal was detected to limit any bias from flies in which multiple blood meals were identified; in a multivariate regression analysis, described below, the number of blood meals detected was included as a covariate. We calculated 95 % Bayesian credible intervals (95 % BCIs) associated with estimated amplification success rates and HBIs using a conjugate Bayesian approach [38]. The Bayesian approach was necessary to construct suitable uncertainty intervals associated with proportions with either small denominators or when the point estimate was close to 0 or 1, and with the derived estimates of vector population sizes as described below.

We estimated the vector population size at each village across each season by rearranging the expression for the blackfly biting rate [39] following [2],

1 $$ V=MBR\times H\times \frac{g}{HBI}, $$

where *V* is the total vector population size; *MBR* is the monthly biting (landing) rate measured as the number of bites per host per month measured per vector collector (see [13]); *H* is the total human population size as recorded in the census conducted; *g* is the mean duration of the period between two consecutive blood meals (taken as the length of the gonotrophic cycle and fixed at 3.5 days [5, 39, 40] expressed in months (3.5 × 12/365 = 0.1151 mo.); and *HBI* is the proportion of blood meals taken on humans calculated as described above. Bayesian credible intervals associated with the estimated vector density were constructed by sampling from the estimated posterior distribution of *HBI*, calculating a value of *V* for each randomly drawn value, and summarising the resulting distribution of *V* by the 2.5 % and 97.5 % percentiles.

We constructed two marginal logistic regression models [41] to explore variation in (1) the amplification success rate and (2) the HBI (this second model was divided into three variants, 2a, 2b and 2c as described below). The coefficients of marginal models can be estimated using generalized estimating equation techniques to account for non-independent (clustered/correlated) data. The potential for dependency arises here because amplification of non-*Simulium* DNA was undertaken on discrete 96-well PCR plates. Hence, amplification success may be more similar among blackflies on the same plate or assay compared with blackflies run on different assays [42]. The response variable for model (1) was defined as whether or not non-*Simulium* DNA was amplified from each blackfly (i.e. a binary variable) and for model (2) as whether or not successfully amplified DNA (i.e. conditional on successful amplification) was of human origin. We calculated so-called sandwich estimators for coefficient standard errors, which were used to construct CIs suitably adjusted for the potential dependence among the data. Given the logistic nature of the models, coefficients correspond to log odds ratios (log ORs) and hence the exponents of these coefficients correspond to ORs.

Both models (1) and (2) included the following as additive covariates: blackfly species; trapping technique; season (wet or dry), year of capture and village. The first variant of the model for the HBI (model 2a) also included, as an additional covariate, the number of identified blood meals per fly (from any vertebrate host; one would expect the chance of detecting human DNA to increase with increasing numbers of blood meals). Two further variants of model (2) were also explored: model 2b, in which the blackfly species covariate was replaced with an indicator of whether they were nominally savannah (*S. damnosum* (*s.s.*)/*S. sirbanum*) or forest/forest-savannah mosaic type (*S. sanctipauli* Pra form; *S. yahense*; *S. soubrense* Beffa form; *S. squamosum* C and *S. squamosum* E), and model 2c, in which the village covariate was replaced with the ratio of the non-human (domestic) mammal population/human population (as estimated from the household, wild bird and mammal surveys presented in Table 1).

## Results

### Household, wild bird and mammal surveys

Table 1 presents the total number of people (listing children - those aged < 18 years - and adults - those aged ≥ 18 years), and domestic animals (chickens, ducks, cattle, sheep, goats, cats, dogs and pigs) as well as the number of different species of wild birds for all study villages, with total numbers of people and domestic animals calculated using the known total number of houses. The number of wild bird species recorded on the transects at each location ranged from 31 to 61, but many additional species were also noted opportunistically (Additional file 1), with 226 species identified altogether. Few other wild animals were recorded; amongst mammals these included red-flanked duiker *Cephalophus rufilatus* (Artiodactyla: Bovidae), a species of small antelope found in western and central Africa - at Asubende; green bush squirrel *Paraxerus poensis* (Rodentia: Sciuridae) at Asubende, Asukawkaw Ferry and Gyankobaa; striped ground squirrel *Xerus erythropus* (Sciuridae: Xerinae), a species of squirrel native to Africa - at Agborlekame and Asukawkaw Ferry; Gambian giant pouched rat *Cricetomys gambianus* (Nesomyidae: Cricetomyinae), a species widespread in sub-Saharan Africa - at Asubende; and African pygmy hedgehog *Atelerix albiventris* (Insectivora: Erinaceidae) at Asukawkaw Ferry. In addition, a tortoise *Kinixys* sp*.* and Agama lizards *Agama agama* were seen at Asubende, with other lizards (Lacertidae) and skinks (Scincidae) present at most sites; a West African green mamba *Dendroaspis viridis* (Squamata: Elapidae), a highly venomous West African snake, was noted at Asukawkaw Ferry.

### Fly collection

A total of 17,300 *S. damnosum* (*s.l.*) flies was collected, of which 6,142 (35.5 %) were caught by vector collectors; 2,207 (12.8 %) were trapped in the human-baited tents; 1,567 (9.1 %) in the cow-baited tents; 7,212 (41.7 %) on Bellec traps, including 3,352 (46.5 % of the Bellec total) from the pilot study in Bosomase during the dry season in 2006 and 172 (1 %) in Monks Wood light traps. In total, 16,478 (95.2 %) blackflies were morphologically identified, of which 5,812 (35.3 %) were dissected for parity in the field, with 4,247 (73.1 %) nulliparous and 1,565 (26.9 %) parous flies. The nulliparous flies were not further analysed for blood meal identification, as *S. damnosum* (*s.l.*) flies feed once per gonotrophic cycle [40] and therefore being host-seeking nullipars it was assumed that they had not previously fed. A further 1,124 flies were donated to the Blackfly Genome Project (https://www.hgsc.bcm.edu/black-fly-genome-project).

### Blood meal identification

The abdomens of 11,107 simuliid females which would have obtained blood meal(s) previously were tested, with blood meals successfully amplified in 3,772 blackflies (yielding a composite unadjusted estimate of the amplification success rate of 33.9 %). In total, 4,847 blood meals were detected giving an average of 1.28 (detectable) blood meals per fly. Of these 3,597 (74.2 %) were identifiable to blood host species. The frequencies of species identified, in descending order, were human (3,004; 83.5 %), porcine (341; 9.5 %), bovine (209; 5.8 %), ovine (15; 0.42 %), canine (1; 0.03 %), caprine (6; 0.17 %) and 21 (0.58 %) of potentially other species including horses, birds and cats. There were 1,251 (25.8 %) unidentified species. A single host species was identified in 2,857 (58.9 %) of the successfully amplified blood meals, of which 75.7 % (2,162/2,857) were of human origin (yielding a composite unadjusted estimate of the HBI of 0.76). Two different blood meal host species were recorded in 761 blackflies (20.2 %), three species in 151 blackflies (4 %), and four blood host species (human, porcine, bovine, and one unknown) were identified from three Bellec-caught flies (0.11 %).

### Amplification success rate (Model 1)

**Fig. 3:**
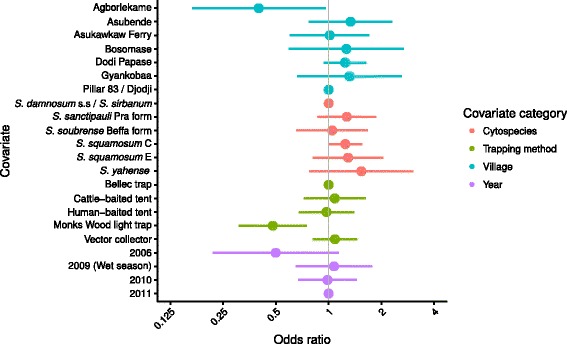
Odds ratios associated with the successful amplification of DNA from *Simulium damnosum* (*s.l.*) Estimates are derived from a multivariate marginal logistic regression model that adjusts for the correlation in amplification success within PCR assays. Horizontal bars represent 95 % confidence intervals constructed from robust sandwich-estimators of the standard error. The vertical line indicates the null effect of a covariate at an odds ratio = 1

The amplification success rate was generally similar among blackfly species, trapping technique, season, year and PCR assay. The ORs and 95 % CIs associated with each of the covariates are shown in Fig. 3. However, blood meals were statistically significantly less likely to be detected in blackflies caught in Agborlekame (OR = 0.40, 95 % CI: 0.17–0.96) and by the Monks Wood light trap (OR = 0.48, 95 % CI: 0.31–0.75) compared with the reference village of Pillar 83/Djodji and the reference trapping technique of the Bellec traps, respectively. Amplification success was lower for flies caught in 2006 compared to the reference 2011 year but this did not reach statistical significance. There was a substantial degree of clustering in amplification success within PCR assays/96-well plates (for blackflies from the same cytospecies; collected from the same village, in the same year and season, i.e. sharing a common set of covariates) as indicated by the estimated correlation coefficient of 0.44.

### Human blood meals (Model 2)

**Fig. 4:**
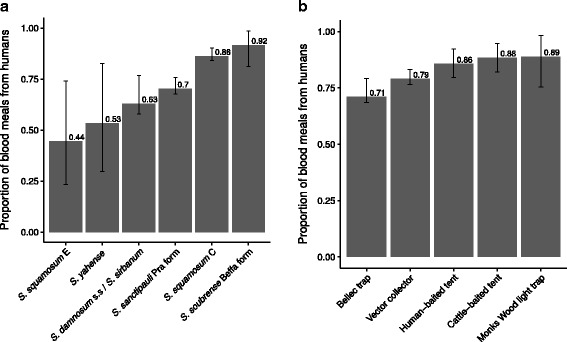
Proportion of amplified blood meals identified as human stratified by *Simulium damnosum* (*s.l.*) cystospecies (**a**) and trapping technique (**b**). DNA was amplified from ovipositing and host-seeking adult female blackflies collected from host-independent traps (Bellec traps and Monks Wood light traps) and host-dependent traps (vector collector, human-baited tent and cattle-baited tent). Data are presented from blackflies with only one detected blood meal and hence the proportions can be interpreted as estimates of the human blood index (HBI). Error bars represent 95 % Bayesian credible intervals

Of blackflies with a detected blood meal, the (univariate) percentages with human blood meals (i.e. the HBIs) by cytospecies and by trapping technique are shown in Fig. 4a and b, respectively. *Simulium soubrense* Beffa form, *S. squamosum* C and *S. sanctipauli* Pra form were the most anthropophagic (HBI = 0.92, 0.86 and 0.70, respectively); *S. squamosum* E, *S. yahense* and *S. damnosum* (*s.s.*)*/S. sirbanum* were the most zoophagic (HBI = 0.44, 0.53 and 0.63, respectively). The estimated (univariate) HBIs for all blackfly cytospecies together, by village, season and trapping technique are given in Table 2. In Table 3 these estimates are further stratified by cytospecies.

**Table 2:** Monthly biting rate, human density, human blood index, vector density and vector to human ratio in study villages, Ghana

Region	Village	Season	MBR	Human host density (*H*)	All trapping techniques			Vector collector-caught flies only		
					HBI (95 % BCI)	*V* (95 % BCI)	*V/H*	HBI (95 % BCI)	*V* (95 % BCI)	*V/H*
Brong-Ahafo	Asubende	Dry Feb 2011	2,061	108	0.65 (0.58, 0.71)	39,603 (35,917–44,480)	367 (333–412)	0.70 (0.56, 0.82)	36,712 (31,280–46,497)	340 (290–431)
	Agborlekame	Dry Feb 2011	775	nd	0.33 (0.01, 0.77)	nd	nd	nd	nd	nd
Volta	Asukawkaw Ferry	Wet Aug 2009	nd	5,459	0.40 (0.18, 0.64)	nd	nd	1.00 (0.23, 1.00)	nd	nd
		Dry March 2010	5,777		0.74 (0.68, 0.80)	4,904,478 (4,570,053–5,340,204)	898 (837–978)	0.67 (0.59, 0.74)	5,424,605 (4,893,429–6,151,412)	994 (896–1,127)
		Dry Feb 2011	5,429		0.99 (0.97, 1.00)	3,453,718 (3,419,227–3,551,323)	633 (626–651)	1.00 (0.98, 1.00)	3,410,288 (3,410,288–3,481,386)	625 (625–638)
	Dodi Papase	Wet Aug 2009	nd	5,254	0.86 (0.56, 1.00)	nd	nd	nd	nd	nd
		Dry March 2010	2,357		0.98 (0.94, 1.00)	1,458,110 (1,431,769–1,533,702)	278 (273–292)	0.99 (0.95, 1.00)	1,445,328 (1,427,147–1,522,330)	275 (272–290)
		Dry Feb 2011	4,371		0.98 (0.94, 1.00)	2,701,299 (2,654,646–2,835,194)	514 (505–540)	1.00 (0.97, 1.00)	2,642,575 (2,642,575–2,741,938)	503 (503–522)
	Pillar 83 /Djodji	Wet July 2009	nd	188	nd	nd	nd	nd	nd	nd
		Dry March 2010	7,171		0.69 (0.63, 0.74)	225,288 (209,692–244,864)	1,198 (1,115–1,302)	1.00 (0.98, 1.00)	155,129 (155,129–159,325)	825 (825–847)
		Dry Feb 2011	9,329		0.91 (0.88, 0.93)	222,982 (217,243–230,326)	1,186 (1,156–1,225)	0.98 (0.96, 0.99)	206,089 (203,430–211,314)	1,096 (1,082–1,124)
Western	Bosomase	Wet Aug 2009	5,481	188	0.73 (0.59, 0.85)	162,045 (139,771–203,188)	862 (743–1081)	0.63 (0.42, 0.83)	187,736 (144,996–290,028)	999 (896–1,127)
		Dry Feb 2010	1,209		0.88 (0.80, 0.95)	29,641 (27,741–33,093)	158 (148–176)	1.00 (0.84, 1.00)	26,154 (26,154–32,700)	139 (139–174)
Ashanti	Gyankobaa	Wet Aug 2009	4,121	274	0.41 (0.37, 0.46)	316,258 (284,223–355,258)	1,154 (1,037–1,297)	0.47 (0.42, 0.53)	276,101 (246,778–312,882)	1,008 (901–1,142)

*Abbreviations:* *MBR* monthly biting rate (no. of bites/person/month) as per vector collector (see [13]), *HBI* human blood index, *H* human host density, *V* vector density, *V/H* vector to human host ratio, *nd* not determined, *BCI* Bayesian credible intervals. In all calculations the length of the gonotrophic cycle (g) was assumed to be 3.5 days, expressed in months (3.5 × 12/365 = 0.1151)

**Table 3:** Human blood index (95 % BCI) by locality, season, trapping method (Bellec and vector collector only) and blackfly species, Ghana

Region	Village	Season	Trapping method	Total *S. damnosum* *(s.l.*)	*S. damnosum* (*s.s.*)*/S. sirbanum*	*S. soubrense* Beffa form	*S. squamosum* ^a^	*S. yahense*	*S. sanctipauli* Pra form
Brong-Ahafo	Asubende	Dry February 2011	Bellec	0.63 (0.54, 0.70)	0.63 (0.54, 0.70)	na	na	na	na
			Vector collector	0.70 (0.56, 0.82)	0.70 (0.56, 0.82)	na	na	na	na
	Agborlekame	Dry February 2011	Bellec	0.33 (0.01, 0.77)	0.33 (0.01, 0.77)	na	na	na	na
			Vector collector	na	na	na	na	na	na
Volta	Asukawkaw Ferry	Wet August 2009	Bellec	0.36 (0.14, 0.60)	0.33 (0.01, 0.77)	1.00 (0.23, 1.00)	0.25 (0.00,0.65)	na	na
			Vector collector	1.00 (0.23, 1.00)	1.00 (0.23, 1.00)	na	na	na	na
		Dry March 2010	Bellec	0.67 (0.43, 0.87)	1.00 (0.23, 1.00)	na	0.58 (0.32, 0.83)	na	na
			Vector collector	0.67 (0.59, 0.74)	0.44 (0.32, 0.58)	0.75 (0.45, 0.97)	0.82 (0.73, 0.90)	na	na
		Dry February 2011	Bellec	1.00 (0.77, 1.00)	1.00 (0.23, 1.00)	1.00 (0.23, 1.00)	1.00 (0.69, 1.00)	na	na
			Vector collector	1.00 (0.98, 1.00)	1.00 (0.92, 1.00)	1.00 (0.43, 1.00)	1.00 (0.98, 1.00)	na	na
	Dodi Papase	Wet August 2009	Bellec	1.00 (0.74, 1.00)	1.00 (0.23, 1.00)	na	1.00 (0.69, 1.00)	na	na
			Vector collector	na	na	na	na	na	na
		Dry March 2010	Bellec	0.67 (0.23, 0.99)	na	na	0.67 (0.23, 0.99)	na	na
			Vector collector	0.99 (0.95, 1.00)	1.00 (0.56, 1.00)	1.00 (0.23, 1.00)	0.98 (0.94, 1.00)	na	na
		Dry February 2011	Bellec	na	na	na	na	na	na
			Vector collector	1.00 (0.97, 1.00)	1.00 (0.74, 1.00)	na	1.00 (0.97, 1.00)	na	na
	Pillar 83/ Djodji	Dry March 2010	Bellec	0.53 (0.46, 0.60)	0.75 (0.35, 1.00)	na	0.53 (0.46, 0.60)	na	na
			Vector collector	1.00 (0.98, 1.00)	1.00 (0.43, 1.00)	na	1.00 (0.98, 1.00)	na	na
		Dry February 2011	Bellec	0.78 (0.70, 0.84)	0.69 (0.44, 0.90)	na	0.79 (0.71, 0.85)	na	na
			Vector collector	0.98 (0.96, 0.99)	1.00 (0.56, 1.00)	1.00 (0.23, 1.00)	0.98 (0.96, 0.99)	na	na
Western	Bosomase	Wet August 2009	Bellec	0.85 (0.68, 0.97)	na	na	na	na	0.85 (0.68, 0.97)
			Vector collector	0.63 (0.42, 0.83)	na	na	na	na	0.63 (0.42, 0.83)
		Dry February 2010	Bellec	0.86 (0.76, 0.94)	na	na	na	na	0.86 (0.76, 0.94)
			Vector collector	1.00 (0.84, 1.00)	na	na	na	na	1.00 (0.84, 1.00)
Ashanti	Gyankobaa	Wet August 2009	Bellec	0.10 (0.04, 0.16)	0.09 (0.00, 0.31)	na	0.25 (0.00, 0.65)	0.00 (0.00, 0.26)	0.10 (0.04, 0.18)
			Vector collector	0.47 (0.42, 0.53)	0.53 (0.30, 0.76)	na	0.50 0.26, 0.74)	na	0.46 (0.40, 0.53)

*Abbreviation: na* not available

^a^ *S. squamosum* C in Pillar 83/Djodji, Dodi Papase and Asukawkaw Ferry; *S. squamosum* E in Gyankobaa

#### Model 2a

**Fig. 5:**
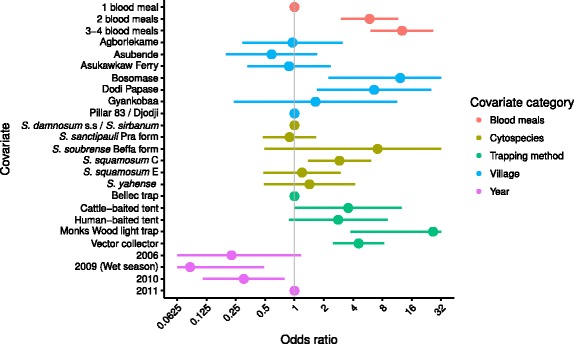
Odds ratios associated with the proportion of successfully amplified DNA that is of human origin from *Simulium damnosum* (*s.l*.) Estimates are derived from a multivariate marginal logistic regression model. Horizontal bars represent 95 % confidence intervals constructed from robust sandwich-estimators of the standard error. The vertical line indicates the null effect of a covariate at an odds ratio = 1

The proportion of blood meals identified as of human origin varied by the number of identified blood meals, blackfly species, trapping technique, village, season and year. The ORs and 95 % CIs associated with each of the covariates are shown in Fig. 5. As expected, the ORs indicate an increasing probability of detecting a human blood meal with the total number of blood meals identified per fly. The probability of detecting a human blood meal was statistically significantly greater in Bosomase (OR = 12.17, 95 % CI: 2.24–32.00) and Dodi Papase (OR = 6.57, 95 % CI: 1.71–25.30) than in the reference village of Pillar 83/Djodji (Fig. 5). Bosomase and Dodi Papase also had large human populations relative to the domestic mammal populations (compare the ratio *M*/*H* of non-human (domestic) mammal/human density in Table 1), suggestive of an influence of the relative abundance of humans on the HBI. The estimates show a statistically significantly greater probability of detecting a human blood meal in *S. squamosum* C (found in Pillar 83/Djodji, Dodi Papase and Asukawkaw Ferry) compared with the reference cytospecies of *S. damnosum* (*s.s*.)*/S. sirbanum* (OR = 2.89, 95 % CI: 1.37–6.10) and apart from *S. sanctipauli* Pra form, the forest/forest-savannah mosaic species (all excepting *S. damnosum* (*s.s*.)*/S. sirbanum*) had point estimates of ORs greater than 1. There was a statistically significantly higher probability of detecting a human blood meal in flies caught in Monks Wood light traps (OR = 26.52, 95 % CI: 3.75–32.00) and by vector collectors (OR = 4.56, 95 % CI: 1.71–25.30) compared with Bellec traps and a statistically significantly lower chance of detecting human blood meals in the 2009 wet season (OR = 0.085, 95 % CI: 0.063–0.49) and the 2010 dry season (OR = 0.30, 95 % CI: 0.11–0.79) compared with the dry season in 2011.

#### Model 2b

When the cytospecies were categorized into savannah or forest/forest-savannah mosaic type, the estimated OR of the latter compared with the former was 2.14 (95 % CI: 0.99–4.63) with a *P* value of 0.05, indicative of borderline statistical significance.

#### Model 2c

When the covariate ‘village’ was replaced by its non-human mammal/human ratio, the estimated OR was 0.77 (95 % CI: 0.51–1.15) indicative of a decline, albeit not statistically significant, in the probability of detecting a human blood meal with a declining relative abundance of humans compared to non-human mammals.

### Non-human mammalian blood meals

**Fig. 6:**
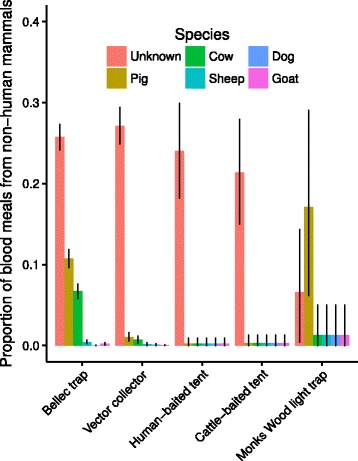
Proportion of amplified blood meals identified as of non-human origin stratified by domestic animal species and trapping technique. DNA was amplified from ovipositing and host-seeking adult female blackflies collected from host-independent traps (Bellec traps and Monks Wood light traps) and host-dependent traps (vector collector, human-baited tent and cattle-baited tent). Error bars represent 95 % Bayesian credible intervals

The percentages of blood meals that were non-human in blackflies caught by trapping technique were: 43.7 % (95 % BCI: 41.9 %–45.5 %) for Bellec traps; 22.4 % (95 % BCI: 10.0 %–35.6 %) for Monks Wood light traps; 21.4 % (95 % BCI: 15 %–27.9 %) for cattle-baited tents; 23.9 % (95 % BCI: 18.1 %–29.8 %) for human-baited tents, and 29.0 % (95 % BCI: 26.7 %–31.3 %) for vector collectors. The majority of non-human blood meals was of unknown origin, porcine or bovine albeit none of the flies caught in the human- or cattle-baited tents had bovine or porcine blood meals identified (Fig. 6).

The percentages of blood meals of bovine origin by blackfly cytospecies were 12.5 % (95 % BCI: 10.5 %–14.6 %) for *S. damnosum* (*s.s*.)*/S. sirbanum;* 3.6 % (95 % BCI: 2.8 %–4.5 %) for *S. squamosum* C and less than 2 % for *S. soubrense* Beffa form, *S. squamosum* E, *S. sanctipauli* Pra form and *S. yahense*. Interestingly, 6.8 % of the blood meals identified in blackflies caught in Pillar 83/Djodji, Bosomase and Gyankobaa were of bovine origin despite no domestic cattle reported in these villages (62.5 % of blood meals identified in blackflies from Agborlekame were bovine but no mammal survey was undertaken there although cattle, pigs and goats were present). Only 1 out of 341 pig blood meals was identified in a blackfly caught in Asukawkaw, the only village to report domestic pig ownership. Pig blood meals were detected in blackflies caught in all other villages with the exception of Agborlekame.

### Vector population size

Using the HBIs and MBRs calculated from the vector collector-caught *S. damnosum* (*s.l*.), estimated vector population sizes ranged from 26,154 flies at Bosomase in the 2010 dry season, to 5,424,605 flies at Asukawkaw in the 2010 dry season. Using HBIs from all analysed flies, estimated vector population sizes varied from 29,641 flies at Bosomase in the 2010 dry season, to 4,904,478 at Asukawkaw in the 2010 dry season (Table 2).

The vector to human ratio (*V*/*H*), calculated using the HBIs estimated from vector-collector caught flies, ranged from 139 at Bosomase in the 2010 dry season to 1,096 at Pillar 83/Djodji in the 2011 dry season. The *V*/*H* values calculated using all analysed flies, peaked at 1,198 at Pillar 83/Djodji in the 2010 dry season. At Bosomase, the only village where we have data for both wet and dry seasons, the *V*/*H* was between five and seven times as high in the wet season (when the monthly biting rate was highest, MBR = 5,481 bites/person/month) and the HBI was lowest (0.63, from vector collectors), as in the dry season (when MBR was 1,209 and HBI was 1.0; Table 2).

## Discussion

Evaluation of the proportion of blood meals taken on humans and of the determinants of host choice by disease vectors is important for understanding (and ultimately manipulating through control interventions) the ecology, evolutionary biology and epidemiology of VBDs [43, 44]. Knowledge of the HBI and how it varies spatially, temporally, by vector species and by vector and/or host diversity and density would enable improved parameterisation of models for the transmission dynamics and control of VBDs [45]. This is particularly important in the context of the elimination efforts spurred by the WHO against infectious diseases of poverty in general and the NTDs in particular [6, 7], many of which are vector borne.

### Methodological considerations

We collected flies using host-dependent (adult host-seeking) and host-independent (ovipositing) blackflies using a range of trapping techniques. Host choice was analysed by collecting flies when attracted to humans or cattle, and through molecular identification of previous blood meals. The propensity to feed on various blood hosts was analysed among *S. damnosum* (*s.l.*) cytospecies, locality, season, trapping technique and human and non-human blood host densities. The HBI values differed between species in an unadjusted analysis across the whole dataset, with *S. squamosum* E and *S. yahense* exhibiting the lowest (0.44 and 0.53, respectively) and the Beffa form of *S. soubrense* the highest (0.92) HBI (Fig. 4a, although the HBIs estimated for *S. squamosum* E and *S. yahense* were also the most uncertain). In the adjusted multivariate analysis (Fig. 5), *S. squamosum* C had a statistically significantly higher OR compared to the savannah members of the *S. damnosum* (*s.l*.) complex (*S. damnosum* (*s.s*.)*/S. sirbanum*), which were indicated as having the lowest HBIs (smallest ORs). In agreement with results presented (for African anophelines) in [11], the OR of identifying a human blood meal decreased with the ratio between non-human and human host density (i.e. with a decreasing relative abundance of humans compared to non-human mammals) as measured in the censuses (Table 1), but this trend was not statistically significant. The increased chance of detecting a human blood meal with an increasing number of blood meals identified is perhaps not surprising. Humans appeared to be the most frequently used blood host (75.7 % of the blood meals identified were of human origin). Therefore, although simuliid host species choice may be sensitive to host availability, a preference for humans seemed to be evident that cannot simply be attributed to random foraging on available host species.

A measure that has been used in host preference studies of disease vectors is the so-called forage ratio (FR, the proportion of blood meals taken from a particular host species divided by its relative abundance within the host community), with values of FR > 1 implying a preference for the host species, FR < 1 indicating avoidance, and FR ~ 1 suggesting randomness [46]. We did not attempt to calculate the FR partly because of the difficulty in conducting accurate (domestic and feral) host censuses (censuses were not conducted at each sampling occasion and consistent transects were not achieved), and partly because it assumes that the presence of a host implies its availability [44].

We had a relatively high success rate in DNA amplification (34 %) and blood meal identification (74 %) from both host-seeking and ovipositing blackflies. The latter will have been collected approximately three to four days after feeding in comparison with resting mosquitoes, which can be collected soon after engorgement [47]. These success rates were comparable to those in previous blackfly blood meal studies, both in onchocerciasis vector [29] and non-vector simuliid species [48, 49]. We found high variation in amplification success among PCR assays (each assay comprising a maximum of 96 samples). That is, successes were correlated, clustered on particular plates. High levels of inter-assay variation have been previously identified in PCR techniques for detecting and quantifying malaria gametocytes in human blood [42]. This can be caused by variations in the quantity of DNA in the sample [50] or a variety of other aspects related to the experimental protocol, including specifics of the primer design [51] and less traceable variations in reagents, equipment and human error. Although we were able to account for this variation in our statistical analysis, it reduced their overall power, decreasing the chance that the effect sizes associated with the variables of interest (e.g. cytospecies, trapping technique) would reach statistical significance.

We did not see a decline in amplification success in host-seeking blackflies in comparison with ovipositing blackflies, which might have been expected due to the increased duration since their previous blood meal (as there would have been a further delay between laying eggs and locating a new host). The lower amplification success in flies caught in Monks Wood light traps may be due to the relatively poor condition of these flies; the traps were only emptied at least 12 h after being set up and most flies were dead and dry prior to fixation, whereas the wild-caught flies were kept alive and cool after capture and the flies in the Bellec traps were partially preserved by the oil within which they were immobilised and kept cool by the splashing river water. The lower (albeit not statistically significant, Fig. 3) amplification success in flies caught in 2006 is probably explained by DNA degradation between the time of capture and storage and the molecular analysis which was undertaken in 2011. Our overall DNA amplification success demonstrates the potential suitability of the techniques applied in our study for use on other vector species, e.g. sandflies [52, 53] and *Culicoides* spp. [54, 55], whose resting sites may be hard to locate, or that are traditionally caught only by host-dependent methods, thereby helping to overcome potential difficulties in investigating their HBIs.

Blood meals were successfully identified from a range of hosts including, primarily, humans, pigs, cattle, sheep, dogs and goats (blood meals from horses, birds and felines were also identified in much lower numbers). The DNA profiling technique used has distinct advantages over ELISA methods [28], as we did not have to anticipate the potential host range. However, 1,251 blood meals remained unidentified and future DNA sequencing would help to elucidate the identity of these host species. We did not detect any chicken blood meals, despite clear profiles being known for this host species, high number of chickens recorded in our domestic animal surveys, and blackflies having been previously recorded to feed on them [20]. In contrast we detected porcine blood meals in all villages except Agborlekame (although only 156 blackflies where caught at Agborlekame), which we did not expect given the published literature [20, 21, 23] and our household surveys. Domestic pigs, *Sus scrofa domesticus*, were only formally counted in Asukawkaw Ferry. However, we subsequently recorded presence of pigs in Abgorlekame - at least 36 - and in Adjalala, Baaya and Beposo, very close to Asubende, as well as in Konongo, near Gyankobaa. We also detected more multiple blood meals than expected (24.3 %) and it is therefore likely that our DNA amplification technique could have picked up previous feeds as well as interrupted ones that would have required completion on more than one host species for satiation at the time of biting; we are assuming gonotrophic concordance, i.e. one blood meal for one batch of eggs rather than the latter requiring multiple, full blood meals [40].

Second to humans, the most commonly detected blood meals were of pigs and cows, which may represent an innate preference, or an effective choice, to feed on larger animals, possibly less defensive, if present. Experimental studies should be conducted to evaluate the potential fitness rewards and costs (nutritional value of blood - reflected in fecundity and fertility - energetic costs of digestion, host defensive behaviour) of the various dietary resources of simuliids in general and simuliid vectors in particular, an area of research that is far more advanced for mosquitoes [44, 56]. Whatever the determinants of host choice, the notion of reducing transmission by manipulating the host species choice of vectors is not new, and perhaps for the *S. damnosum* species complex in West Africa our results could provide novel opportunities for parasite and/or vector control, through zooprophylaxis with pigs and cattle [24, 57], as well as potential improvement of traps, baited with pig or cattle odours, and/or with large silhouettes and high CO_2_ production, particularly for the savannah members of the *S. damnosum* (*s.l.*) complex. However, the potential benefits gained from zooprophylaxis are unlikely to be linearly associated with increased animal abundance, and likely to be dependent on blackfly mortality whilst host searching, as well as on the impact of any changed husbandry practices on other vector fitness components [44, 56, 58]. Earlier studies at Djodji (opposite Pillar 83), on the Togolese side of this breeding site on what is known as the Wawa river in Ghana (Gban-Houa river in Togo), showed that although both human and cattle bait successfully attracted *S. sanctipauli* Djodji form (now eliminated [59]) and *S. squamosum* C, both cytospecies were highly anthropophagic [19]. Thus, they may be more likely to contribute to maintaining transmission of *O. volvulus* than less anthropophagic species and to be less susceptible to zooprophylaxis or even potentially benefited by zoopotentiation [56, 60].

Although bovine blood meals were detected in 6 % of the blackflies, none of these were caught in the cattle-baited tents. Vector collector-caught flies had a similar proportion of human blood meals compared with flies caught in the tents (Fig. 5), despite vector collectors being far more attractive to host-seeking flies than either the cow- or human-baited tents (the number of flies in the human-baited tent catches was considerably lower than in the vector-collector catches [13, 18]). Hence one could argue that these results disagree with the hypothesis that host choice by haematophagous arthropods may be influenced by prior foraging experience, causing them to learn which hosts are most successfully fed upon [44]. However, our study design was not optimal to investigate this question, as the baited tents were not as successful in attracting host-seeking flies as it was hoped. It is noteworthy that we cannot discount that blood meals might not reflect only past feeds but rather that some inadvertent contamination during collection may have occurred (e.g. by flies succeeding in getting a human blood meal upon landing on the vector collector, prior to capture). However, the similar ORs associated with the probability of detecting a human blood meal in blackflies caught by vector-collectors, human- and cow-baited tents suggest that contamination was minimal.

### Modelling and transmission considerations

Vectorial capacity encapsulates the entomological components of the basic reproduction ratio, *R*_0_, of a VBD [61, 62], and in mathematical models of VBD transmission, the biting rate per vector on humans enters as a squared term, becoming highly influential [2, 44]. It is, therefore, important that the HBI is estimated accurately and that macro- and micro-geographical, secular, seasonal, habitat- and capture site-related, and host density-influenced heterogeneities are investigated and quantified [45]. Transmission indices such as the Annual Biting Rate (ABR), Annual Infective Biting Rate (AIBR), and Annual Transmission Potential (ATP) quantify composite measures of exposure and transmission intensity as they are based on net biting rates, but in full transmission models the biting rate per vector on humans is also entered separately [2]. Our data provide the first rigorous body of evidence (by molecularly-based blood meal analysis) that HBI varies between different members of the *S. damnosum* (*s.l*.) complex*.* Up until now, evidence of zoophagy and anthropophagy has been more circumstantial, informed by the ratio of human to non-human filarial larvae found in flies [63, 64], fly species that were collected in Bellec traps but not on human attractants at the same sites and times [65], or based on observations of resting flies with very small sample sizes [21], and a few host-dependent studies [19, 22].

HBI varied between simuliid cytospecies, and although the majority of all blood meals had been taken on humans, *S. squamosum* C and *S. soubrense* Beffa form showed greater anthropophagy than *S. damnosum* (*s.s.*)*/S. sirbanum*, *S. sanctipauli* Pra form, and *S. yahense* (compare ORs in Fig. 5), supporting previous indications of high anthropophily (attraction to humans) in *S. squamosum* [19], and zoophily in some populations of *S. sirbanum* [65, 66] (note that very few *S. squamosum* E were caught resulting in the most uncertain estimate of HBI, Fig. 4a). The Beffa form of *S. soubrense* has previously been reported to be highly anthropophilic and an efficient vector of *O. volvulus* [17]. Such anthropophagy is supported here (although the OR is not statistically significant), and although the villages where we collected *S. soubrense* Beffa form in great numbers - those in the Volta Region - have received long-term community-directed treatment with ivermectin (CDTI) - and we observed very low *O. volvulus* infection levels in the flies [18] - the high biting density of *S. soubrense* Beffa form and its high HBI could be a risk factor for infection resurgence, particularly as transmission is still occurring in the region [18].

An effect of season and year of capture on the HBI was detected. The OR associated with detecting a human blood meal was statistically significantly lower in the 2009 wet season (Fig. 5; note that wet season data were collected in 2009 only and hence we cannot disentangle the two). Seasonal changes in host choice by disease vectors have been reported, with these shifts probably due to seasonal changes in host availability, host reproductive phenology and climatic conditions among other explanations [44, 67–69]. More difficult to explain is the lower chance of detecting human blood meals in the 2006 and 2010 dry seasons (compared with the 2011 dry season). Presuming that the wet season is at least partly driving the lower ORs recorded for 2009, one explanation is that the dry season of 2010 was unusually wet. However, the dry season in 2011 was wetter than the 2006 and 2010 dry seasons (Additional file 2: Figure S1). Since the timing of CDTI in relation to seasonal vector–human contact is an important programmatic consideration, our results can inform modelling studies that take into account transmission seasonality to guide optimal frequency and timing of microfilaricidal treatment for onchocerciasis elimination [12].

The more anthropophagic a vector species, the lower the magnitude of the transmission breakpoint [10] - the parasite density below which the infection would die out - and hence the more difficult it is to achieve elimination. In addition, the threshold biting rate for endemic persistence (for *R*_0_ ≥ 1) would also be lower [9], so a lower vector density would be needed to maintain endemic transmission (possibly helping to explain persistence of transmission under situations of low biting rate). We have reported active transmission in Asubende, Bosomase and Gyankobaa [18], the former in particular with a history of vector control during the OCP and > 24 years of CDTI (twice per year since 2009 [18, 33]). In this formerly highly hyperendemic community (76 % baseline prevalence), where *S. damnosum* (*s.s*.)/*S. sirbanum* prevail, HBI was 0.63–0.70, with MBR = 2,061 bites/person/month, and a vector to host ratio of 340–367 flies per human. Although the vector in this location has moderate anthropophagic tendencies and biting rates, a possible explanation for the persistence of transmission is the statistically significantly higher rate of skin repopulation by *O. volvulus* microfilariae reported in [31] for individuals in Asubende despite biannual treatment, raising concerns over how best to control transmission in this area, and highlighting potential issues such as decreased ivermectin efficacy [70]. Complementary and/or alternative treatment strategies such as focal (larvicidal) vector control, potentially zooprophylaxis, and anti-wolbachial treatment of those individuals with persistent microfilaridermia may accelerate progress towards elimination [71].

In Bosomase, we had previously recorded higher onchocerciasis transmission in the wet season than during the dry season [18], despite the HBI being generally lower in the wet season (Table 2). Larvicidal vector control may therefore be best suited to help to reduce transmission in these forest areas. At Gyankobaa, where we had also reported high levels of transmission, we recorded a low HBI which translated into a vector to human host ratio of ~1,000 blackflies per person (Table 2); these two quantities are inversely linked according to eqn (1). Gyankobaa has never received vector control, and our results indicate that this may be a useful intervention that would help to lower vector population density and reduce transmission.

In contrast with the above mentioned communities, we had reported very low *O. volvulus* infection levels in blackfly populations at Asukawkaw Ferry, Dodi Papase and Pillar 83/Djodji (which had pre-control infection prevalence levels ranging from 67 to 76 % [18]), indicating that the current (annual) CDTI strategy is working well. However, here we report high HBIs across these three Volta region villages, highlighting that if CDTI were halted prematurely, the risk of infection rebound could be substantial, particularly as the parasite densities corresponding to transmission breakpoints are likely to be very low [8].

Our initial hypotheses included that, at its simplest, effective host preference would vary with the relative abundance of non-human and human hosts, and *R*_0_ would exhibit only weak non-linear responses as a result of the biting rate per fly on humans entering as a squared term [11]. This conjecture was supported by our data, only that the negative relationship between HBI and the non-human to human host ratio did not reach statistical significance. However, we also hypothesized that effective host preference might also vary with the ratio of vectors to hosts [2, 72]. This was predicted to be a strongly non-linear relationship, with *R*_0_ varying non-linearly with vector abundance in addition to varying with the ratio of host abundances [2, 43]. Control strategies seeking to reduce vector abundance would have very different impacts on disease transmission depending on the shape of the relationship between *R*_0_ and the vector to host ratio. The same would apply to control through zooprophylactic manipulation of the abundance of alternative hosts [3]. However, as evidenced by the results summarised in Table 2, there was no clear relationship between the independently measured HBI and MBR, and the decreasing relationship between HBI and *V*/*H* is simply a function of eqn (1). Therefore, our results do not support the conjecture of a vector or host density-dependent contact rate in onchocerciasis transmission.

The discrepancy between the conclusions reached by the theoretical enquiry of [2] and by the analysis of empirical data presented in this paper could be because in the former it was assumed that the observed mean microfilarial load in the community represented the sum total of the exposure events that lead to parasite acquisition, with the HBI being estimated to fit this worm burden given a recorded biting rate, whereas in this paper the MBR represents a maximal measure of exposure to blackfly bites, unlikely to represent effective vector–host encounters. Jacobi et al. [73] showed that determining an actual index of exposure (according to bioclime - forest or savannah - host age and sex, occupation and length of stay at site of blackfly biting, clothing, and defensive behaviour) provided a better measure of realised fly engorgement on human blood and a better relationship with individual microfilarial load. These authors estimated that only about 30 % of vector-human host encounters would effectively contribute to onchocerciasis transmission in Cameroon [73].

Overall, our blood meal analysis results indicated an average HBI of 0.63 for savannah members of the *S. damnosum* (*s.l*.) complex in Ghana, lower than the 0.96 assumed by the ONCHOSIM model - which is parameterised using Asubende data [8] - but higher than the 0.3 used in the EPIONCHO model for Cameroon settings [12, 32, 39]. Basáñez et al. had indicated a hypothetical HBI range between 0.01 and 0.84 [2], and between 0.1 and 0.99 [9] for localities in Burkina Faso, Cameroon and Côte d’Ivoire, estimated using EPIONCHO’s precursor [39]. Additional file 2: Figure S2 presents an informal EPIONCHO validation exercise for West African savannah settings using 0.67, the average HBI reported in this paper for *S. damnosum* (*s.s*.)/*S. sirbanum* in Asubende. For forest/forest-savannah mosaic vector species (i.e. all species excepting *S. damnosum* (*s.s*.)*/S. sirbanum*), our blood meal analyses indicate an average HBI of 0.78, which together with the very high vector competence and vectorial capacity of these species [10, 15, 17, 19, 73], may result in having to reach lower transmission breakpoints for onchocerciasis elimination in areas where these species prevail.

### Limitations

Due to early attempts at the start of the project in the 2009 rainy season to trial alternative, more ethical methods to measure biting rate (rather than using standard OCP vector-collector methods), we have very limited data on biting rates during this time and, therefore, cannot directly compare vector abundance between seasons within villages, except at Bosomase, where we have data for both seasons and recorded greater fly numbers in the wet than in the dry season. However, we cannot extrapolate these trends to the other villages because the availability and productivity of blackfly breeding sites is not always positively correlated with rainfall [74–76], neither is fly survival. In fact, we have shown that fly survival, estimated from parity rates, is lower in the wet season, although biting rates may be higher [5, 13].

The lack of a clearer relationship between the type of domestic animals recorded in our surveys and the blood meal species identified in the corresponding village, particularly for cattle and pigs, suggests that blackflies are likely to locate hosts and obtain blood meals across an area greater than just the village near breeding sites. However, as discussed earlier, presence of a potential blood host does not necessarily equate with it being ‘available’, and calculation of FR values (not attempted here) are fraught with difficulties [44]. Although the hypothesis was that vector breeding sites would ‘serve’ their proximate communities, the foraging spatial niche of blackflies may be substantially wider, a consideration that needs to be taken into account when delineating onchocerciasis transmission zones.

Detecting past blood meals in host-seeking or ovipositing flies as done in this study did not allow a distinction to be made, when encountering multiple host species, between interrupted feeds contributing to the same (immediately previous) gonotrophic cycle and the relics of blood meals obtained during past gonotrophic cycles. This limits our understanding of the relationship between the findings of multiple blood meals and the interval between two consecutive feeds (which is important for the calculation of the biting rate per fly on hosts), the possibility that gonotrophic concordance may not always apply, and any heterogeneity in transmission potential, including *O. ochengi* as well as *O. volvulus*, from flies which had fed on both bovines and humans (essential to the operation of cross-immunological protection between these two species [24, 25, 57]). However, we had reported that all the *Onchocerca* larvae we identified molecularly from infected/infective flies during this study were *O. volvulus* [18]. The overall number of multiple feeds may have been underestimated as the technique we used cannot differentiate between a single meal from one host and multiple meals from multiple hosts of the same species; besides, smaller (weaker) blood meals are less likely to be detected. Therefore, when measuring the HBI, we will have included multiple human feeds, but excluded them when they were identified together with another host species. This may have slightly biased the HBIs downwards as suggested by the higher OR of detecting a human blood meal with increasing blood meal number.

## Conclusions

This study has demonstrated that molecular analysis of prior blood meals, leading to blood host identification, is feasible in a range of epidemiologically important cytospecies of the *S. damnosum* (*s.l.*) complex, vectors of *O. volvulus* in savannah and forest regions of Ghana and West Africa. Members of this species complex have heterogeneous trophic preferences, and important covariates explaining variation in the HBI included capture site/method, and season. None of these heterogeneities are adequately captured by current onchocerciasis transmission models [2, 5, 8, 9, 12, 32]. We did not, however, find strong evidence supporting the hypothesis of vector and/or host density dependence in the contact rate for onchocerciasis models that would warrant modification of current formulations of the infection reproduction ratio [2, 43]. Such formulations do not presently consider the relative contribution to transmission of different vector ensembles when multiple members of the *S. damnosum* (*s.l*.) complex are present in a particular location simultaneously or during different times of the year [13, 18].

Based on the findings of our previous papers [5, 13, 18] and the results of this study, we echo the conclusions of [45], which although drawn from a review and comparison of (prevalence-based) mosquito-borne disease models, also apply to infection intensity frameworks for filariases in general and human onchocerciasis in particular, and which call for an expansion of VBD modelling to include, among others, heterogeneous vector biting, spatial heterogeneity and temporal variation in the transmission process if such models are to be truly useful to inform public health policy and practice.

## Abbreviations

ABR, annual biting rate; AIBR, annual infective biting rate; ATP, annual transmission potential; BCI, Bayesian credible interval; CDTI, community directed treatment with ivermectin; CI, confidence interval; ELISA, enzyme-linked immunosorbent assay; FR, forage ratio; *g*, duration of gonotrophic cycle; *H*, human density; HBI, human blood index; *M*, non-human mammal density; *M/H*, non-human (domestic) mammal to human host ratio; MBR, monthly biting rate; MDA, mass drug administration; NTD, neglected tropical disease; OCP, Onchocerciasis Control Programme in West Africa; OR, odds ratio; *R*_0_, basic reproduction ratio; *s.l*., *sensulato*; *s.s*., *sensu stricto*; *V*, vector density; *V/H*, vector to human host ratio; VBD, vector-borne disease; WHO, World Health Organization

## Additional files

Additional file 1: Bird species recorded in the study. (XLSX 24 kb) Additional file 2: Rainfall data for study years and informal validation of EPIONCHO using the value of HBI estimated for the savannah members of the *Simulium damnosum* (*s.l*.) complex. (DOC 94 kb)
