# Supplementary material for: Onchocerciasis transmission in Ghana: the human blood index of sibling species of the Simulium damnosum complex
Source: Parasit Vectors. 2016 Aug 5;9:432. doi: 10.1186/s13071-016-1703-2 (PMC4975878; doi:10.1186/s13071-016-1703-2)
Supplement: Additional file 2: — Rainfall data for study years and informal validation of EPIONCHO using the value of HBI estimated for the savannah members of the Simulium damnosum (s.l.) complex. (DOC 94 kb) [file 13071_2016_1703_MOESM2_ESM.doc]

**Additional File 2**

**Rainfall data for study years and informal validation of EPIONCHO using the value of HBI estimated for the savannah members of the *Simulium damnosum* s.l. complex.**

In an attempt to explain the lower chance of detecting human blood meals in the 2006 and 2010 dry seasons (compared to the 2011 dry season), and presuming that the wet season could at least partly drive the lower odds ratio (OR) recorded for 2009 (Figure 5 of main text), we conjectured that one possible explanation is that the dry season of 2010 was unusually wet. We explored this hypothesis informally by plotting monthly recorded rainfalls in the region (Figure S1). However, we found the converse; the dry season in 2011 was wetter than the 2006 or 2010 dry seasons.

**Figure S1.** **Monthly rainfall and year of blackfly collections.** The rainfall data come from a meteorological station located in Kumasi, near to the sampled villages. Shaded rectangles indicate the dates of sampling during the dry seasons of 2006 (January–February), 2010 (February–March) and 2011 (January–March) and the wet season in 2009 (July–September).

We informally validated EPIONCHO [1,2] against published [3] pre-control data on microfilarial prevalence (the gold standard measure of infection prevalence) and annual vector biting rates from Burkina Faso, Cameroon and Côte d’Ivoire (Figure S2).


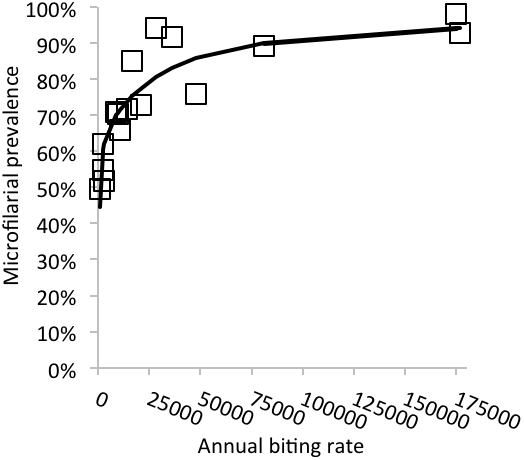


**Figure S2. Informal validation of EPIONCHO against pre-control microfilarial prevalence data from Burkina Faso, Cameroon and Côte d’Ivoire.** The solid line represents the predicted endemic equilibrium prevalence of *Onchocerca volvulus* skin microfilariae in the human population for different values of the annual biting rate (the number of vector bites per person per year) assuming a human blood index of 0.67 for the savannah members of the *Simulium damnosum* *s.l.* complex (the average value of HBI in the locality of Asubende).

We found that EPIONCHO matched the pattern in the data very closely by modifying the human blood index (the proportion of blood meals taken on humans) of the blackfly vectors. The proportion of blood meals taken on humans was increased from one third [3] to two thirds (for the savannah members of the *Simulium damnosum* species complex) according to results presented in the main text.

**References**

1. Filipe JAN, Boussinesq M, Renz A, Collins RC, Vivas-Martinez S, Grillet ME, Little MP, Basáñez MG. Human infection patterns and heterogeneous exposure in river blindness. Proc Natl Acad Sci U S A. 2005; 102(42):15265-15270.

2. Turner HC, Walker M, Churcher TS, Osei-Atweneboana MY, Biritwum NK, Hopkins A, Prichard RK, Basáñez MG. Reaching the London Declaration on Neglected Tropical Diseases goals for onchocerciasis: an economic evaluation of increasing the frequency of ivermectin treatment in Africa. Clin Infect Dis. 2014; 59(7):923-932.

3. Basáñez MG, Boussinesq M. Population biology of human onchocerciasis. Philos Trans R Soc Lond B Biol Sci. 1999; 354(1384):809-826.
